# Laser Fragmentation Synthesis of Colloidal Bismuth Ferrite Particles

**DOI:** 10.3390/nano10020359

**Published:** 2020-02-19

**Authors:** Simon Siebeneicher, Friedrich Waag, Marianela Escobar Castillo, Vladimir V. Shvartsman, Doru C. Lupascu, Bilal Gökce

**Affiliations:** 1 Technical Chemistry I and Center for Nanointegration Duisburg-Essen (CENIDE), University of Duisburg-Essen, Universitaetsstr. 7, 45141 Essen, Germany; simon.siebeneicher@uni-due.de (S.S.); friedrich.waag@uni-due.de (F.W.); 2 Institute for Materials Science and Center for Nanointegration Duisburg-Essen (CENIDE), University of Duisburg-Essen, 45141 Essen, Germany; marianela.escobar@uni-due.de (M.E.C.); vladimir.shvartsman@uni-due.de (V.V.S.); doru.lupascu@uni-due.de (D.C.L.)

**Keywords:** nanoparticles, size reduction, picosecond, ray tracing, multiferroic

## Abstract

Laser fragmentation of colloidal submicron-sized bismuth ferrite particles was performed by irradiating a liquid jet to synthesize bismuth ferrite nanoparticles. This treatment achieved a size reduction from 450 nm to below 10 nm. A circular and an elliptical fluid jet were compared to control the energy distribution within the fluid jet and thereby the product size distribution and educt decomposition. The resulting colloids were analysed via UV-VIS, XRD and TEM. All methods were used to gain information on size distribution, material morphology and composition. It was found that using an elliptical liquid jet during the laser fragmentation leads to a slightly smaller and narrower size distribution of the resulting product compared to the circular jet.

## 1. Introduction

The perovskite bismuth ferrite (BFO) unites several interesting material properties and attracts strong research interest as a multiferroic material [1,2]. It combines ferroelectricity and magnetism at room temperature, which enables interesting applications in data storage and communication technology. However, since the material is usually antiferromagnetic as a bulk, lattice distortion needs to be introduced into the crystal to generate a total magnetic moment unequal to zero. BFO also exhibits the ferroelectric photovoltaic effect [3,4], which allows triggering magnetic spin states by light illumination. The unique combination of material properties of BFO could enable new generations of data storage [5] and communication technologies [6] in the future. Furthermore BFO attracts attention as a photocatalyst [7].

One way to synthesize magnetisable BFO is the epitaxial growth of thin films [8]. The synthesis of nanoparticles represents another possibility to introduce magnetization into BFO [9,10]. However, compared to thin films, nanoparticles can be applied in a broader field of applications.

Shetty et al. synthesized phase-pure but relatively large (average grain size of 61.6 nm) BFO nanoparticles by wet chemical precipitation combined with calcination in 2002 [11]. The achievable minimum particle size could be further reduced to the range of 10 to 20 nm by applying wet chemical synthesis approaches followed by calcination within works of Ghosh et al. [9,12]. As summarized and reviewed by Goa et al., single step synthesis processes without additional calcination or annealing could be demonstrated and BFO nanoparticles of different morphologies became accessible [7]. The synthesis of crystalline BFO nanoparticles with diameters below 10 nm has so far remained unattained.

Laser fragmentation of colloidal particles is an alternative method that is used to downsize oxide particles to diameters in the low single-nanometre range [13,14,15,16]. In contrast to other size reduction techniques, laser fragmentation is a wear-free method. High-intensity light radiation transfers the energy into the process and initializes the size reduction thermally or by Coulomb explosion, depending on the laser intensity and the electronic response of the irradiated material [16]. However, it was also found that the extreme conditions of high temperatures and pressures during the size reduction can cause unexpected reactions involving the surrounding media. For example, the ablation of a glassy carbon target in tetrahydrofuran (THF) leads to the formation of carbon nanoparticles presumably covered in polymerized solvent, leading to a surprising colloidal stability [17]. While this effect is beneficial, the liquid can impair particle quality as well. Ablation of a cobalt target in water shows indications for oxidation which in this case worsens the magnetic properties (i.e., decreases coercivity). Oxidation did not occur when utilizing ethanol as media [18]. Another unwanted effect is the phase decomposition of ferrite particles [19]. This decomposition was partly attributed to an inhomogeneous laser energy distribution during irradiation of the colloidal educt.

In this study, we investigated the laser fragmentation of colloidal BFO particles. Changes in particle size and morphology during laser fragmentation were studied by transmission electron microscopy. Decomposition effects and loss of crystallinity were analysed by selective area electron diffraction and X-ray diffraction. In addition, the geometry of the laser-irradiated colloid jet was manipulated from circular to elliptical to obtain a more homogeneous distribution of laser energy in the irradiated colloid. The energy distribution was modelled for circular and elliptical jet geometries by a ray tracing approach and the influence of the geometry variation on structural properties of the product was investigated experimentally.

## 2. Materials and Methods

### 2.1. Synthesis of Educt Powder

Synthesis of the bismuth ferrite powder was carried out using a nitrate based route published by Ghosh et al. [9]. 15 mmol of each $Bi{\left(NO_{3}\right)}_{3}\cdot 5H_{2}O$ (Alfa Aesar, Kandel, Germany) and $Fe{\left(NO_{3}\right)}_{3}\cdot 9H_{2}O$ (VWR-Chemicals, Hannover, Germany) were dissolved in 2 N $HNO_{3}$ and 30 mmol tartaric acid (Merck, Darmstadt, Germany) were added. This solution was then dried at 80 °C and pre-calcined at 150 °C on the hotplate. The resulting fluffy powder was mortared and sintered at 600 °C for two hours. After confirming phase purity by XRD, the powder was wet-milled in ethanol with $ZrO_{2}$-balls of different size until it was possible to easily disperse it in water using ultrasonication.

### 2.2. Laser Fragmentation Setup

**Figure 1:**
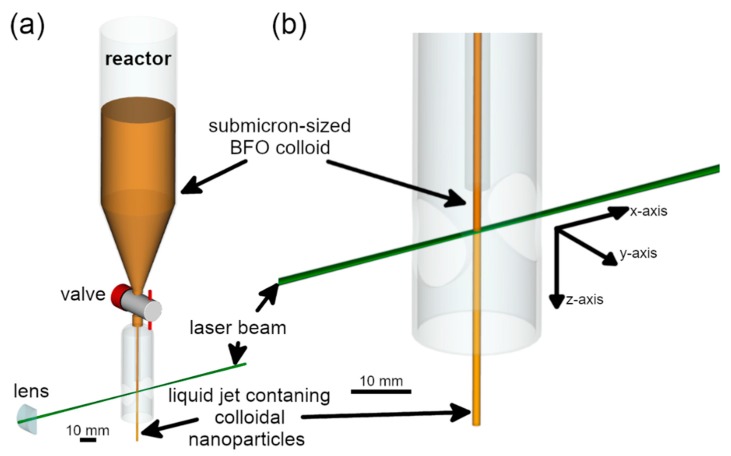
Illustration of the laser fragmentation setup. (**a**) Overview of the setup that consisted of a reactor ending in a valve to control the liquid flow. The laser beam (only z-axis) was focused onto the liquid jet. (**b**) Zoom-in of the spot where the colloid jet and the laser beam intersect.

Laser fragmentation of the dispersed BFO powder was performed using a liquid jet flow setup (Figure 1) with additional precise jet velocity control and utilization of circular and elliptical orifices. The elliptical orifices were produced in house by laser-drilling an elliptical hole into an aluminium sheet.

For precise positioning of the elliptical water jet, a micrometre-controlled positioning and measuring system was developed. With the help of a red laser pointer, a beamline perpendicular to the fragmentation beamline was set up. By focusing this laser in front of the water jet, a sharp shadow is cast on a screen allowing for positioning of the water jet with a precision of about 100 µm. In case of the elliptical orifice, the fragmentation laser was positioned in the first repetition of the elliptical jet pattern and the size of the elliptical profile was verified using the laser pointer setup (see Supplementary Materials). Before performing the final experiments, a fluence study was performed to find the laser fluence that would have the greatest effect on the extinction spectrum of the colloid. On two laser systems, fluences ranging from 32 to 1500 mJ/cm^2^ were compared (details can be found in the Supplementary Materials, Figures S2 and S3). The highest fluence was chosen as this also produced the strongest change in the UV-VIS extinction spectrum.

Fragmentation was carried out in a way that every particle was irradiated once by the high fluence part of the laser pulse in z-direction. We defined the area of the Gaussian energy profile in z-direction, which contained 50 % of the pulse energy, as the high fluence region. In our setup, the laser beam was focused to a spot with a diameter of 79 µm (z-direction). The aforementioned high fluence area covered about 39.5 µm in the centre of the spot. With a volume flow of 1.64 mL/s through an elliptical orifice of 0.518 mm^2^ this resulted in 1.16 pulses per particle, slightly above the one pulse-per-particle region as a precaution to hit every particle at least once (also confer Table 1 and Table 2).

### 2.3. Analytical Instruments

XRD-measurements were carried out with a Panalytical Xpert pro MPD diffractometer (Malvern Panalytical GmbH, Kassel, Germany) utilizing $CuK_{\alpha 1}/K_{\alpha 2}$-radiation at 40 kV and 40 mA. Detailed analysis of the diffractograms was performed via Rietveld refinement within the software MAUD (Luca Lutterotti, version 2.7, Trento, Italy). Scanning electron microscopy (SEM) measurements were done on a FEI Quanta 400f (Thermo Fischer Scientific, Gräfelfing, Germany), transmission electron microscopy (TEM) and selected area diffraction (SAED) as well as energy-dispersive X-ray (EDX)measurements on a JEOL 2200FS (JEOL, Freising, Germany).

## 3. Results and Discussion

### 3.1. Laser Fragmentation Using a Circular Colloid Jet

**Figure 2:**
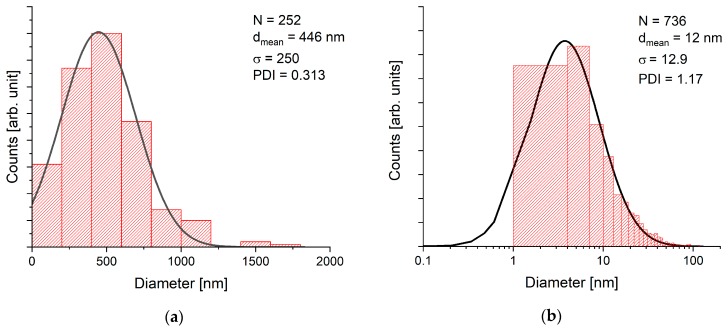
Size distribution of BFO before (REM) (**a**) and after (TEM) (**b**) irradiating the colloids with similar laser parameters utilizing a circular water jet.

**Figure 3:**
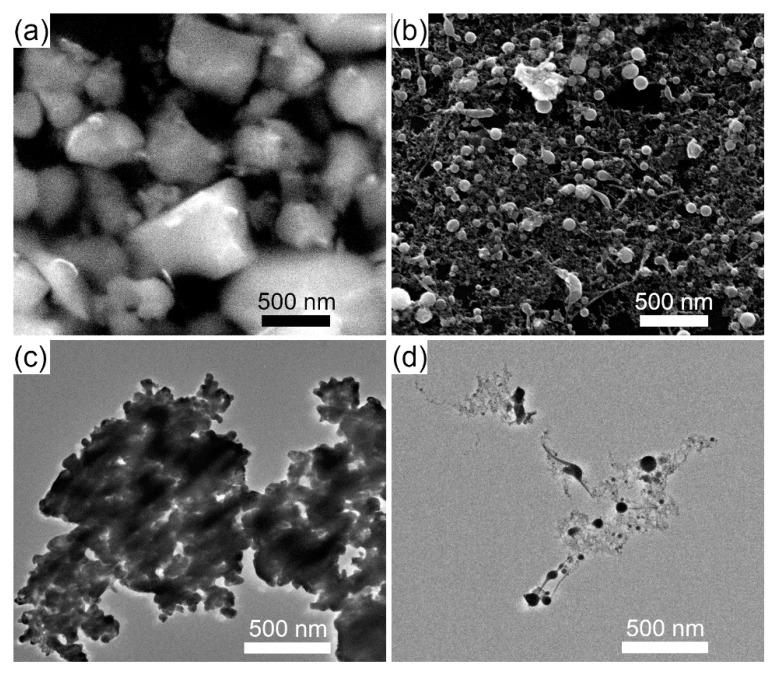
Representative SEM images of the BFO educt (**a**) and the product after irradiating the circular jet (**b**). Representative TEM images of the BFO educt (**c**) and the product from the circular jet irradiation (**d**).

Laser fragmentation of the BFO powder educt was performed by irradiating a circular liquid jet (Figure 1) with a diameter of 1300 µm. As illustrated in the distributions in Figure 2 and the electron microscopy images in Figure 3, a strong size reduction took place during the laser fragmentation. The educt colloid with a mean particle size of 450 nm was reduced by the fragmentation process to a colloid with mean particle diameter of 12 nm. Educt particles were only rarely found in the fragmentation product. As figure of merit the polydispersity index (PDI) was calculated by dividing the variance of the fit by the mean diameter (PDI$\equiv \frac{\mathrm{variance}}{{\mathrm{mean}}^{2}}=\frac{\sigma^{2}}{d_{mean}{}^{2}}$).

However, different species of product particles showed up like very small, low-contrasted particles and larger, high-contrasted spheres (cf. Figure 3b,d). Interestingly, some larger product particles seemed to have tail-like structures and larger non-spherical structures appeared. Those structures may represent frozen melts, which could be due to fast cooling of laser-irradiated educt particles. However, their presence in the product also demonstrates that areas of low laser fluences exist in the irradiated colloid during the fragmentation of the BFO colloid.

As observed in a previous study, the focusing of the laser beam by the circular liquid jet as well as its reflection lead to different fluence regimes in the irradiated colloid [19]. Even though we performed the experiment at a high average fluence, the optical interaction of the Gaussian laser beam with the circular liquid jet could have contributed to the observed results.

### 3.2. Fluence Simulation in Laser-Irradiated Colloid Jets of Different Geometries

**Figure 4:**
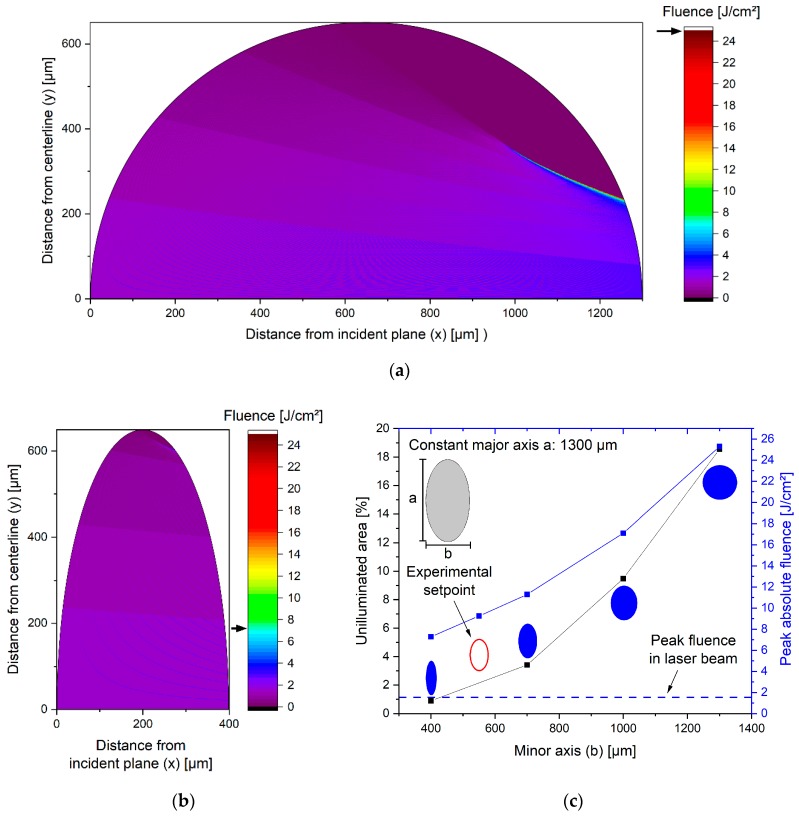
Simulation of energy distribution in a circular (**a**) and an elliptical (**b**) fluid jet. Arrows show highest fluence in jet. The evaluation of the simulation (**c**) shows the two main criteria for homogenized energy-distribution: peak absolute fluence as indicator for “hot spots” and the fraction of pixels not receiving any radiation. A more elliptical jet will have less unilluminated area and a lower peak fluence. Note that in our experiments the major axis of the elliptical jet was about 1200 µm (minor axis 550 µm) due to manufacturing constraints; values for peak fluence (11 J/cm²) and unilluminated area (2.8%) stay comparable.

Because every fluid jet has a curved outer surface, an incident light beam will get refracted at the fluid/air interface. This leads to a focusing of the laser into the fluid jet and to some areas not being illuminated at all, while in places where multiple rays overlap a strong increase in fluence can be observed (cf. Figure 4a). The fluid interface would have to be flat and perpendicular towards the laser beam to omit this effect entirely. In pulsed laser melting of colloidal boron particles, Ishikawa and Koshizaki found a higher sphericity factor for particles melted in a guided flow compared to particles melted in a circular liquid jet [20]. The guided flow was realised by injecting a liquid jet into the 1-mm wide slit between two quartz plates. Laser radiation entered the flow on one of the non-enclosed sides. A less bent interface of the guided flow between the plates could be expected and may have contributed to the more efficient melting. However, at an absolute size in the range of < 1 mm the surface tension of most fluids will lead to a curvature of the fluid surface. In case the colloid should be truly irradiated in just one pass through the reactor, the next best option is to utilize the surface tension of the fluid jet in favour of the process. An elliptical water jet already approximates the flat interface condition, if the eccentricity is as close to unity as possible. To show that an elliptical jet will homogenize the fluence distribution within the jet, a raytracing-based model was developed which allowed calculation of the energy density within the liquid jet.

In µm-steps an incident ray is rastered from the middle over the top half-major-axis of an ellipse with given dimensions. At the point of incidence, the inclination angle is calculated and with the help of Snell’s refraction law the refraction angle is obtained. With this, the beam path of the refracted beam is constructed. Higher order reflections are not calculated in this simulation. Knowing the beam paths and the incident energy distribution (Gaussian laser intensity profile is assumed) each incident ray is assigned an initial fluence. The initial fluence of the refracted beams is reduced by the reflected fraction which is calculated using Fresnel’s law assuming unpolarized light. The refracted beams are attenuated by their travel in the opaque liquid (absorption coefficient of liquid and diluted particles are taken into account) and lose energy. The lost energy is calculated using Lambert Beer’s law. At the end of these calculations two arrays are obtained, one containing data for every ray’s absolute position and another containing the fluence at that position. The absolute position is rounded up to generate a definite position for later addition. Finally, a superposition of every ray is performed, again assuming unpolarized light, and an image containing the energy density of every pixel is obtained.

As illustrated in Figure 4c, a reduction of the minor axis b of the ellipse leads to an improved energy distribution within the fluid jet. When a more elliptical jet (i.e., smaller minor axis b) is introduced the unilluminated area decreases, as well as the peak fluence within the jet. The simulation shows a decrease in unilluminated area by 95% (from 18.5% to 0.9%) while the peak fluence decreases by up to 71% (from 25 J/cm^2^ to 7 J/cm^2^).

Both the reduction in unilluminated area and the decrease in peak fluence can be beneficial. A reduction in unilluminated area is expected to allow a higher fraction of particles to be irradiated, thereby increasing the product particle fraction. Also, better illumination will reduce the number of iterations needed to irradiate every particle contained in the colloid. This translates into better colloid control due to the ability to precisely irradiate every particle with a given energy.

The reduction of the peak fluence may help, if the fluence for fragmentation needs to be close to the breakdown fluence. As demonstrated by Mafuné et al. and Werner et al. for gold, an increase in laser fluence leads to a higher fraction of small (<5 nm) particles [21,22]. Lau et al. showed for ZnO that an increase above the fluid breakdown fluence negates this trend and only moderate fragmentation efficiency can be achieved [23]. Improving the jet geometry may help with the limiting effect of breakdown fluence. In the circular jet, focusing leads to the formation of areas where the fluence is about 17× higher than the peak laser fluence. Accordingly, those areas will reach the breakdown fluence much earlier than the surrounding liquid. In an elliptical jet those areas receive only about 5× peak laser fluence allowing for greater laser fluences to be applied.

### 3.3. Comparison of Laser Fragmentation Using Circular and Elliptical Colloid Jets

In order to verify the hypothesis that an elliptical water jet will homogenize the fluence distribution, laser fragmentation of the dispersed educt powder was carried out in an elliptical jet (550 × 1200 µm). This section compares the results of the experiments to those resulting from the fragmentation using the circular jet (1300 × 1300 µm). During both fragmentation experiments, similar incident laser fluences (~1.5 J/cm^2^) were applied. The number of pulses per particle was kept around 1.14 to ensure every particle being hit at least once.

**Figure 5:**
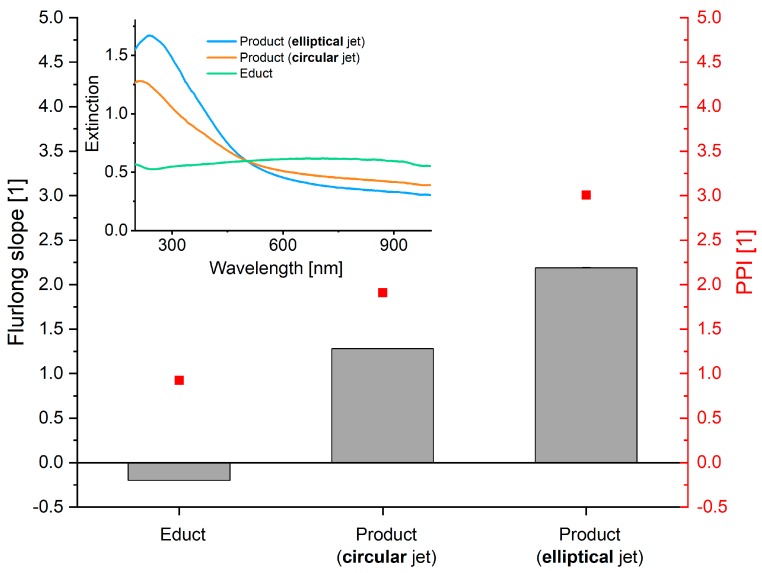
Furlong slope and primary particle index of educt and product colloids after laser fragmentation in circular and elliptical water jets show significant decrease in particle size. Inset shows corresponding UV-VIS spectra.

A first characterization of the colloids was performed via UV-VIS-spectroscopy. As an indication of size distribution the Furlong slope [24] and primary particle index (PPI) [25] were calculated from the obtained spectra. The Furlong slope is retrieved by normalizing the spectra to 450 nm and measuring the negative exponential slope $\left(E=\frac{E(\lambda )}{E(450nm)};S=-\frac{d\log(E)}{d\log(\lambda )}\right)$. A high Furlong slope value is indicative for a high fraction of small nanoparticles. Another figure of merit also showing indications for a high fraction of small particles is the PPI defined as the fraction of extinction at 380 nm and 800 nm $\left(PPI=\frac{E(380nm)}{E(800nm)}\right)$. The results, shown in Figure 5, allow for the conclusion that a particle size reduction of the educt particles was possible with the circular as well as the elliptical jet. Furthermore, both the Furlong slope and the PPI suggest that fragmentation in the elliptical jet was more efficient, leading to a stronger increase of both indicators compared to the circular jet.

**Figure 6:**
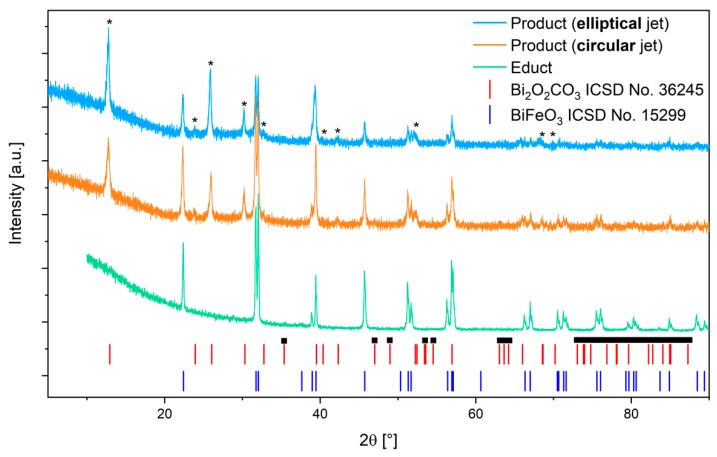
XRD-Measurements of educt and product BFO particles. Asterisks indicate phases possibly belonging to bismuth carbonate, boxes indicate reflections not found that also belong to the carbonate.

Powder-XRD was performed as a second indicator for particle size and to get structural information on the laser-irradiated material (Figure 6). Crystalline phases of the educt and product powders were identified, and average crystallite sizes were determined. Rietveld-refinement of the educt particle diffractogram reveals a single BFO phase with average crystallite size of 240 ± 5 nm. The most striking characteristic of the diffractograms of the products from both fluid jet geometries is the appearance of additional peaks not belonging to BFO. A closer inspection of the additional peaks suggests that they do not belong to iron-rich phases (FeOOH, Fe_2_O_3_, Fe_3_O_4_, Fe_3_C, Fe_3_(CO)_12_) or another BFO phase (Bi_2_Fe_4_O_9_). The best fit is bismuth carbonate (Figure 6 asterisks), but many peaks that should also appear with bismuth carbonate are not found (Figure 6 boxes), which results in unsuccessful Rietveld-refinement (using ICSD No. 36245). Still this phase appears to be the most likely. Interestingly, D’Angelo et al. observed the formation of bismuth carbonate sheets in their comprehensive study on the laser irradiation of colloidal bismuth oxide nanoparticles in aqueous environment [26]. In addition, according to Sylvestre et al. even gold-carbonate-complexes form during the laser ablation of the inert metal in water [27]. The authors suspected dissolved atmospheric CO_2_ to take part in the formation, which would also make sense in our case of using a non-enclosed liquid jet. As in D’Angelo’s study the bismuth carbonate formed sheets, it may be hypothesized that this is also the case for our sample. As sheets are likely to form a common orientation upon XRD-sample preparation, this may result in diffraction peaks being suppressed and would explain the missing peaks in our diffractogram.

Interestingly, the ratios of the areas of the first diffraction peaks of both product phases (carbonate-like at 13° and BFO at 22°) significantly differ for the particles produced by the circular and elliptical jet. Irradiation of the circular jet produced a product in which the area-ratio (carbonate/BFO) is around 1.25 while in the elliptical jet a ratio close to 2.8 indicated a much larger content of carbonate phase. This might be explained by the larger fraction of irradiated fluid in the elliptical jet. The formation of bismuth carbonate may be linked to the BFO decomposition upon melting. As we applied high enough fluences for fragmentation, melting is very possible and could be observed after irradiation of BFO in the circular jet (cf. Figure 3b,d). If the formation of the carbonate-phase is a direct consequence of irradiating BFO at high enough fluences, the higher illuminated fraction in the elliptical jet will yield more carbonate than in the circular jet.

Regardless of the unassigned diffraction peaks, a refinement of the BFO phase was performed to evaluate average crystallite sizes in the product powders. In case of the circular jet, the crystallites appeared to be 80 ± 2 nm in size while in case of the elliptical jet a reduction to 66 ± 1.5 nm was observed. The lower average crystallite size indicates that a larger quantity of particles was fragmented in the elliptical jet. This result is in accordance with the UV-VIS measurements that also indicated a larger quantity of smaller particles in samples from the elliptical water jet.

**Figure 7:**
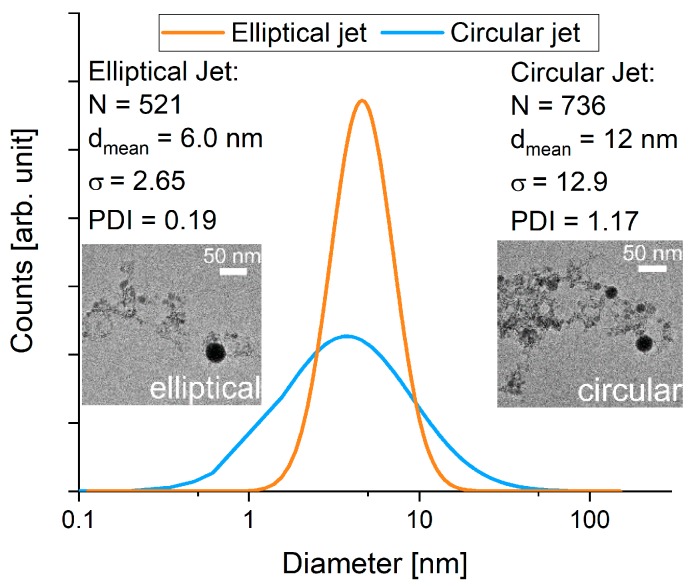
Size distribution (TEM) after irradiating the colloids with similar laser parameters utilizing either a circular or an elliptical water jet.

To further quantify the effect of the changed fluid jet geometry, the product size distributions were also compared using TEM (cf. Figure 7). While the circular jet was able to produce particles with a mean diameter of 12 nm, the elliptical jet allowed for a stronger size reduction, giving a mean diameter of 6 nm. The difference in size distribution width is more pronounced with the elliptical jet, producing a narrower distribution with a standard deviation of 2.7 compared to 13 in the circular jet. This is also reflected in the increased polydispersity index. Fragmentation in the circular jet produces a broad size distribution with a PDI of 1.17 while the elliptical jet can produce a narrow distribution with a PDI of 0.19.

As expected, both jets lead to a reduction in particle size indicating a high enough laser fluence above the fragmentation threshold of the material. Unexpectedly, the TEM size distribution showed much smaller particles than XRD. This observation can be explained when the images are screened visually for crystalline particles. Hardly any crystalline particles could be observed in samples from both jet geometries. This indicates that a large fraction of the product contained amorphous particles, which did not attribute to the XRD-diffractograms, explaining the much larger particle size measured in XRD. Even though TEM and XRD give different size information on the analysed particles, similar trends were found for the products of the circular and elliptical jet. The elliptical jet appears to produce slightly smaller particles with a narrower size distribution compared to particles produced in the circular jet.

**Figure 8:**
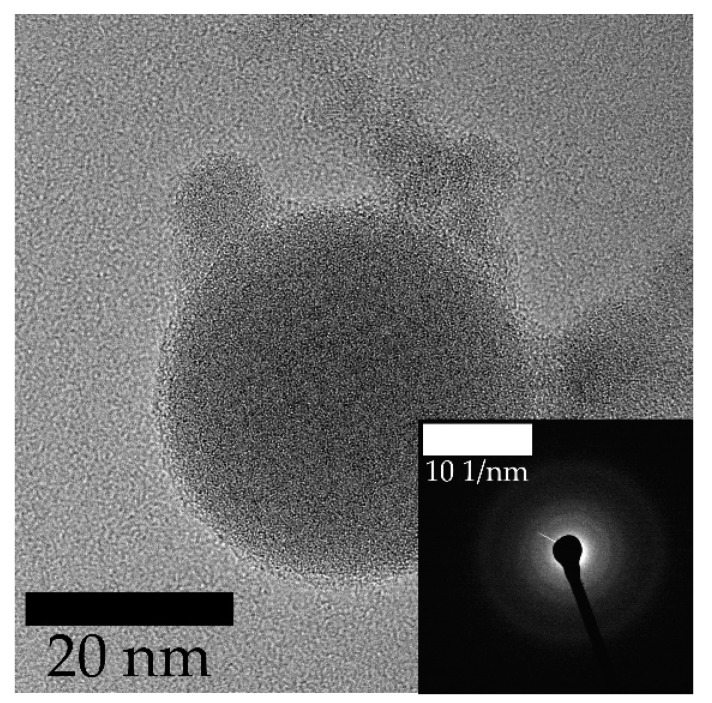
TEM image of a representative particle ensemble synthesized by laser fragmentation with an elliptical jet and the corresponding SAED pattern.

As mentioned above, hardly any crystalline particles could be found in the laser fragmented samples and a comparison revealed no clear differences between the fluid jet geometries in this regard. An exemplary fragmented particle is shown Figure 8. The particle ensemble in this figure caused no SAED-pattern proving its amorphous nature. This observation leads to the assumption that the strong laser irradiation initiates a decomposition of the educt material.

**Figure 9:**
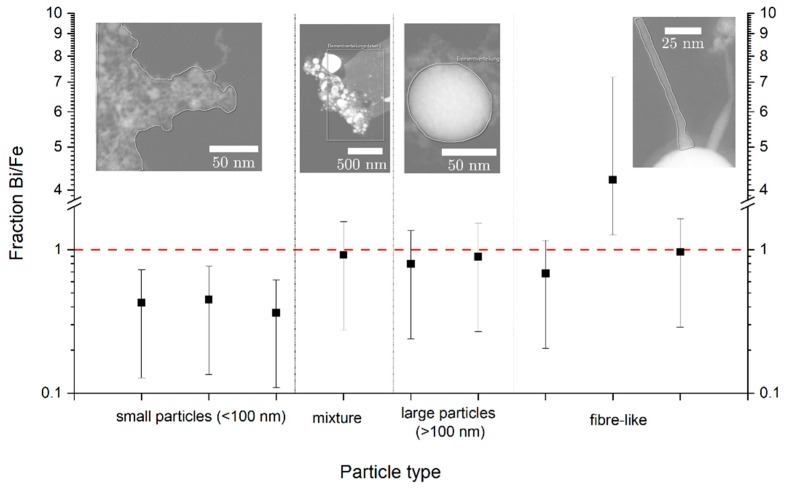
Metal fractions from predominant particle morphologies. Both jet geometries show the same types and Bi/Fe fractions and are combined in this figure. The plotted error bars represent an estimated relative error of 70% which is expected for the experimental conditions.

To get a more detailed insight into the material composition, an analysis via EDX was performed. Because no differences between samples from the circular and elliptical jet could be identified in EDX, all information on material composition was classified into four categories depending on particle morphology and analysed area. The categories that were defined contained small particles (defined as <100 nm), large particles (defined as >100 nm), mixtures and fibre-like particles (cf. Figure 9). This allows for a discussion based on product morphology which seems to have a much larger impact on composition than the fluid jet geometry as also suggested by similar XRD-patterns. The Bi/Fe-fraction was chosen as indicator of the particle composition. In a stoichiometrically correct BiFeO_3_, the Bi- to Fe-ratio should be equal to unity. The amount of oxygen was not quantified as the error coming from the sample holder was too significant for a viable analysis. The plotted error bars in Figure 9 represent an estimated relative error of 70% which can be expected for the presented measurements where the total signal from the nanoparticles lies between 1% and 10% of the total EDX-Signal. Figure 9 shows that only mixtures and particles larger than 100 nm had a Bi/Fe-ratio close to unity. In case of the fibre-like particles, only one sample fulfilled this criterion. All particles smaller than 100 nm have a Bi/Fe fraction below one, indicating decomposition during laser fragmentation. The low Bi/Fe ratio indicates iron-rich compounds which contrasts the XRD-findings showing bismuth-rich compounds. This may be explained by the signal origin analogous to the size findings, as the amorphous iron-rich particles won’t show up in XRD. Surprisingly, no bismuth-rich crystalline phases could be identified in TEM which would be expected. The results show that a decomposition of BFO under strong laser irradiation is very likely to take place. This is probably due to the very high temperatures and pressures applied during the laser fragmentation process [28,29]. All fragmented particles (i.e., particles <100 nm) should have been exposed to the extreme environmental conditions and thereby originate from the laser fragmentation. Particles larger than 100 nm and fibres are likely to have passed through a low-fluence region or not have been irradiated at all, which leads to a Bi/Fe-ratio close to the bismuth ferrite educt. One outlier in the fibre-like particles can be found that contains much more bismuth than all other particles. Presumably this is a species where the remaining bismuth from other morphologies has accumulated. Looking at the mixture, a composition close to unity is expected, because a high ratio of the signal-intensity will originate from large particles which have a ratio close to unity. From this can be concluded that while fragmentation of the educt powder in an elliptical jet leads to small particles and even improves the resulting size distribution, it also results in a decomposition of the educt material that is in EDX indistinguishable from the product powder produced by the circular jet.

## 4. Conclusions

Laser fragmentation of BFO colloids results in a strong size reduction of the educt powders from 450 nm to below 10 nm as shown by UV-VIS, XRD and TEM analysis. After obtaining an inhomogeneous product from the fragmentation in the circular colloid jet, we applied an elliptical jet to homogenize the fluence distribution in the laser-irradiated colloid volume. From the ray tracing simulation, a reduced peak fluence and an increased illuminated colloid volume were expected. As indicated by our results, a decrease in the peak fluence by applying an elliptical colloid jet could not prevent a phase decomposition of BFO. Contrarily, the phase decomposition of BFO seems inevitable. The elliptical jet leads to an even stronger decomposition and a reduced overall particle size, proving the increased interaction volume of laser beam and colloid.

It may be hypothesized that the decomposition of BFO upon picosecond laser irradiation is not triggered by high fluence areas as appear in a circular fluid jet. This leaves other pathways towards decomposition. Firstly, it is possible that chemical reactions of superheated, but stoichiometrically intact, bismuth ferrite with the surrounding solvent plasma lead to secondary products. Or secondly, BFO is itself unstable at the extreme conditions and decomposes into its constituents which subsequently react to form the observed products. We also tested fragmentation in propylene carbonate, however, did not find significant differences to the samples produced in water (see Supplementary Materials). Future studies should consider the unclear decomposition behaviour of bismuth ferrite when processed by laser fragmentation. As almost exclusively spherical particles were found in TEM, it seems possible that even at a laser pulse length as low as 10 ps some thermal influence leading at least to melting is involved in the particle formation mechanism. For melting to occur, the laser pulse length needs to be in the range or longer than the electron–phonon relaxation time ($\tau_{e-ph}$) as for example described by Link et al. [30] for the case of nanosecond laser pulses. For femtosecond pulses, Zhang et al. reported surface melting of silicon ($\tau_{e-ph}$= 350 fs) when using a 457 fs pulsed laser [31]. In the case of BFO, $\tau_{e-ph}$ is reported to be 0.7 ps [32] which is much shorter than the applied pulse length in our experiments. Therefore, a decomposition following melting and evaporation of the ferrite appears possible. A quick quenching of a disordered liquid or gas phase in the surrounding liquid could also explain the mainly amorphous nature observed in TEM. Using short femtosecond pulses may deliver the energy quick enough to lead to Coulomb explosion, in which only a small fraction of energy is transferred to the lattice, possibly keeping the stoichiometry and crystal structure intact. Apart from laser pulse length, future studies should also involve tailoring educt material and solvent to find an ideal chemical environment for BFO synthesis. For example, in pulsed laser deposition (PLD) of BFO thin films it is customary to use a slightly super-stoichiometric BFO to cope with the volatility of bismuth and to tightly control oxygen partial pressure [33,34,35]. In our study we found a too low bismuth content of the smallest particles, which may be counteracted by increasing bismuth content in the educt material. A matching of oxidation potential of the surrounding liquid may help to mimic oxygen partial pressure in PLD and give another important tuning parameter for phase purity.

## Figures and Tables

**Table 1:** General laser parameters.

Parameter	Value
Wavelength (m)	532
Beam quality factor (M^2^)	1.2
Pulse length (ps)	10
Beam diameter (mm)	3

**Table 2:** Detailed process parameters.

Parameter	Elliptical Jet	CIRCULAR Jet
Laser power (W)	83.7	48.4
Pulse frequency (kHz)	92.95	54.52
Pulse energy (µJ)	900	888
Peak fluence of incident Gaussian beam (J/cm^2^)	1.54	1.52
Focus length (mm)	100	100
Distance from lens back (from housing) (mm)	103.4 (100.96)	103.4 (100.96)
Colloid volume (mL)	196	195
Orifice dimension (major axes) (µm)	550 × 1200	1300 × 1300
Flow time (s)	119	91
Pulse per particle	1.16	1.13

## Supplementary Materials

The following are available online at https://www.mdpi.com/2079-4991/10/2/359/s1, Figure S1: Measurement of elliptical jet and comparison to measured elliptical orifice, Figure S2: UV-VIS extinction spectra of BFO dispersions fragmented with fluences of 32, 129 and 174 mJ/cm^2^ and different passages. Absolute differences in the graphs are caused by concentration differences. 0p always shows the corresponding educt powder, Figure S3: UV-VIS extinction spectra of BFO dispersions fragmented with 320, 450 and 1500 mJ/cm^2^. Absolute differences in the graphs are caused by concentration differences. A fluence of 1500 mJ/cm^2^ leads to a Furlong slope of 2.2, Figure S4: Furlong slope and primary particle index of educt and product colloids in water and propylene carbonate after laser fragmentation in circular and elliptical water jets shows significant decrease in particle size. Inset shows corresponding UV-VIS spectra.
