# Supplementary material for: Laser Fragmentation Synthesis of Colloidal Bismuth Ferrite Particles
Source: Nanomaterials (Basel). 2020 Feb 19;10(2):359. doi: 10.3390/nano10020359 (PMC7075302; doi:10.3390/nano10020359)
Supplement: Supplementary file 1 [file nanomaterials-10-00359-s001.pdf]

nanomaterials

Supplementary

Laser Fragmentation Synthesis of Colloidal Bismuth Ferrite Particles

Simon Siebeneicher [1], Friedrich Waag [1], Marianela Escobar Castillo [2], Vladimir V. Shvartsman [2], Doru C. Lupascu [2] and Bilal Gökce [1,*]

[1] Technical Chemistry I and Center for Nanointegration Duisburg-Essen (CENIDE), University of Duisburg-Essen, Universitaetsstr. 7, 45141 Essen, Germany; simon.siebeneicher@uni-due.de (S.S.); friedrich.waag@uni-due.de (F.W.)

[2] Institute for Materials Science and Center for Nanointegration Duisburg-Essen (CENIDE), University of Duisburg-Essen, 45141 Essen, Germany; marianela.escobar@uni-due.de (M.E.C.); vladimir.shvartsman@uni-due.de (V.V.S.); doru.lupascu@uni-due.de (D.C.L.)

* Correspondence: bilal.goekce@uni-due.de

Received: 07 February 2020; Accepted: 13 February 2020; Published: 19 February 2020

1. Verification of elliptical jet geometry

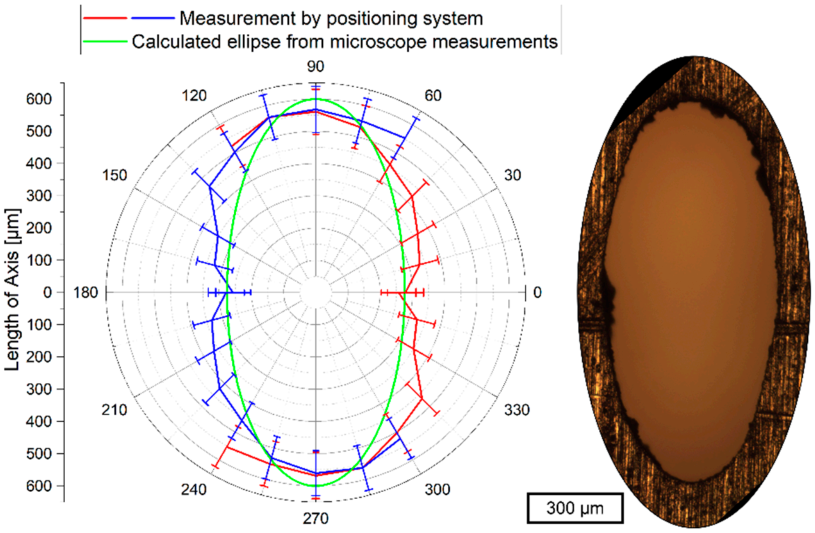

Figure S1. Measurement of elliptical jet and comparison to measured elliptical orifice.

When using a non-circular orifice, the jet geometry repeats several times perpendicular and parallel to the orifice until eventually approaching circular geometry. The exact nature of the jet is determined by jet velocity and orifice dimension and additionally the intersection volume by horizontal positioning relative to the incident fragmentation laser. Our fragmentations where always performed in the maximum of the first repetition after the orifice and the get geometry was verified with the positioning laser. As shown in Figure S1 the dimensions of the liquid jet are in good agreement with the used orifice dimensions.

2. Fluence studies

Preliminary studies on the fragmentation of BFO included studies with different laser fluences. On an EKSPLA Atlantic 532-1064 system (EKSPLA, Vilnius, Lithuania) experiments were performed with a wavelength of 532 nm a pulse length of 10 ps and laser peak fluences of 32, 129 and 174 mJ/cm². The educt dispersion was guided through the orifice multiple times to increase total energy input into the system. Every complete run is called a passage (p).

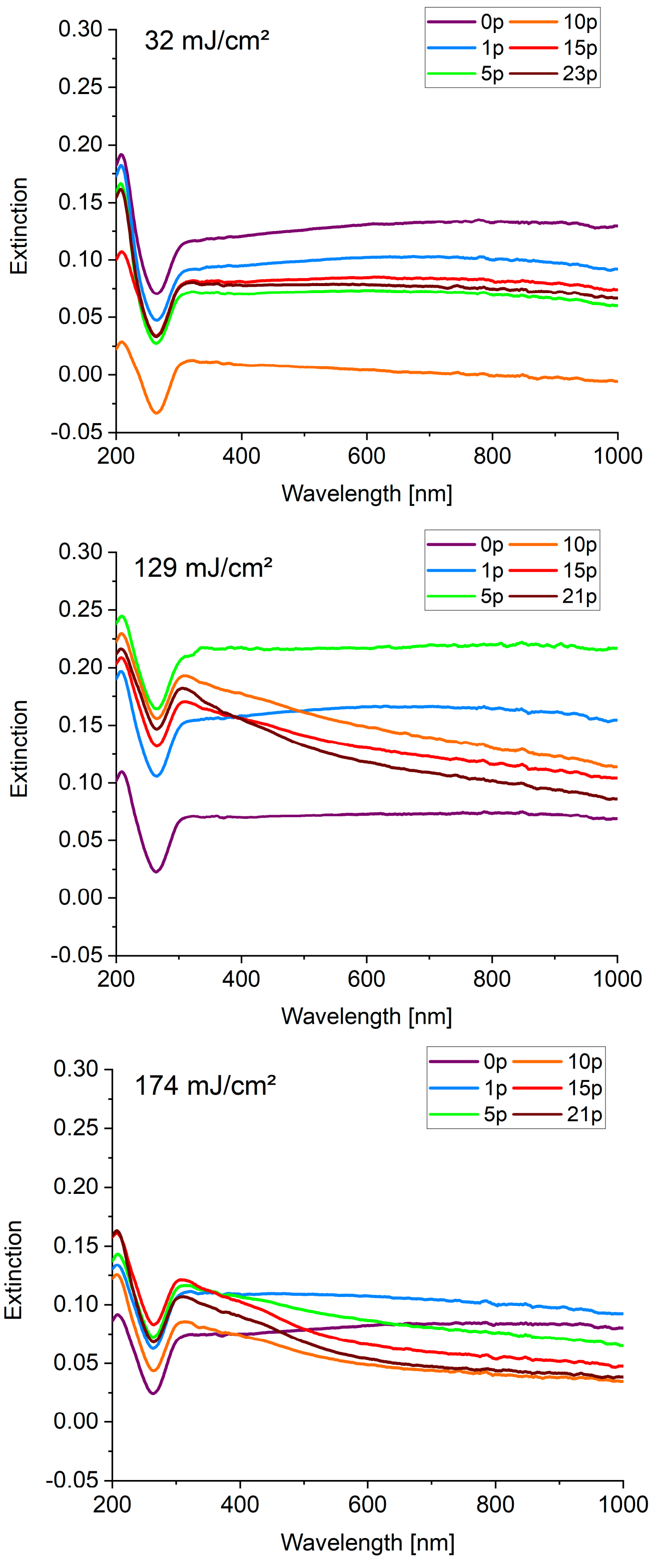

Figure S2. UV-VIS extinction spectra of BFO dispersions fragmented with fluences of 32, 129 and 174 mJ/cm² and different passages. Absolute differences in the graphs are caused by concentration differences. 0p always shows the corresponding educt powder.

As shown in Figure S2 fragmentation of bismuth ferrite with comparably low fluences only shows changes starting at 129 mJ/cm² and only after the colloid has passed 10 times (10p) through the laser path. A higher fluence of 174 mJ/cm² shows a similar trend with differences now becoming visible after 5 passages. The most energy input into the system at 174 mJ/cm² and 21 passages leads to a Furlong slope of 1.24, which was interpreted as incomplete fragmentation. Therefore we changed

the laser system to the one presented in the manuscript and picked the fluence that showed the greatest effect after one passage to be compared to the circular jet (Figure S3).

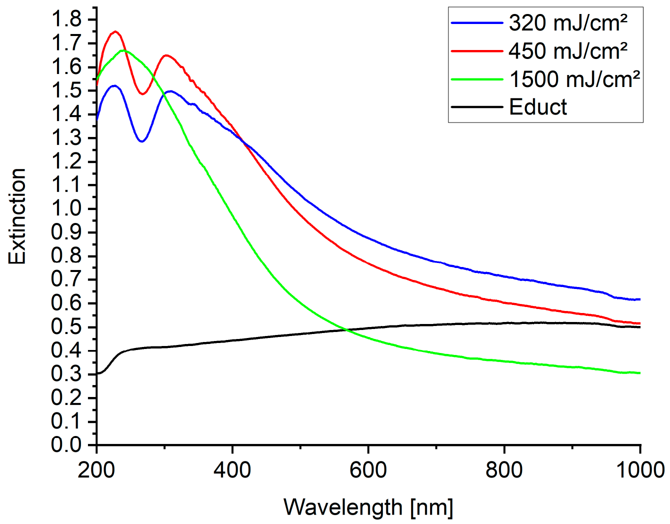

Figure S3. UV-VIS extinction spectra of BFO dispersions fragmented with 320, 450 and 1500 mJ/cm². Absolute differences in the graphs are caused by concentration differences. A fluence of 1500 mJ/cm² leads to a Furlong slope of 2.2.

3. Solvent influence

Apart from water, the low vapour pressure solvent propylene carbonate was used as medium in the elliptical jet. It was found that with a PPI of 2.2 and a Furlong slope of 1.4 it is comparable to the results obtained for the water sample (Figure S4).

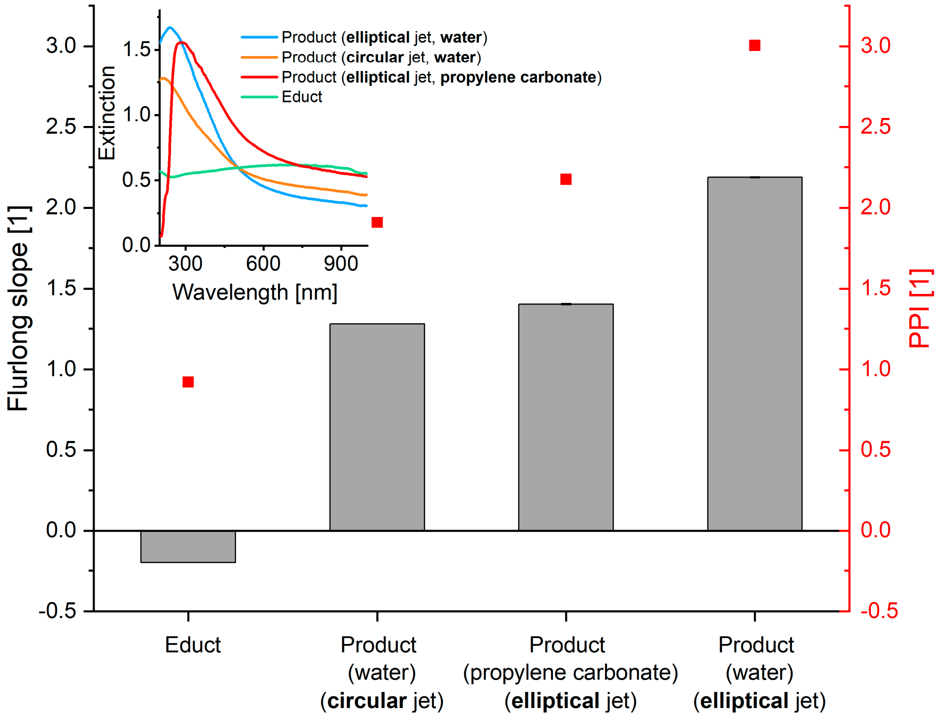

Figure S4. Furlong slope and primary particle index of educt and product colloids in water and propylene carbonate after laser fragmentation in circular and elliptical water jets shows significant decrease in particle size. Inset shows corresponding UV-VIS spectra.

In a TEM-EDX analysis of the sample irradiated in propylene carbonate no differences compared to the water samples could be found.

The sample preparation for XRD required centrifugation of the colloid because of the extremely low vapour pressure of propylene carbonate. This led to size separation as the smallest particles did not sediment after strong centrifugation. XRD-analysis of the sediment revealed only bismuth-ferrite, but due to the preparation limitations (including a high proportion of amorphous fraction) it cannot be excluded that decomposition products (i.e. bismuth carbonate) were present in the colloid.

© 2020 by the authors. Submitted for possible open access publication under the terms and conditions of the Creative Commons Attribution (CC BY) license (http://creativecommons.org/licenses/by/4.0/).
